# The study of organelle DNA variability in alloplasmic barley lines in the NGS era

**DOI:** 10.18699/VJ19.589

**Published:** 2020-02

**Authors:** M.G. Siniauskaya, A.M. Makarevich, I.M. Goloenko, V.S. Pankratov, A.D. Liaudanski, N.G. Danilenko, N.V. Lukhanina, A.M. Shimkevich, O.G. Davydenko

**Affiliations:** Institute of Genetics and Cytology of the National Academy of Sciences of Belarus, Minsk, Belarus; Institute of Genetics and Cytology of the National Academy of Sciences of Belarus, Minsk, Belarus; Institute of Genetics and Cytology of the National Academy of Sciences of Belarus, Minsk, Belarus; Institute of Genetics and Cytology of the National Academy of Sciences of Belarus, Minsk, Belarus; Institute of Genetics and Cytology of the National Academy of Sciences of Belarus, Minsk, Belarus; Institute of Genetics and Cytology of the National Academy of Sciences of Belarus, Minsk, Belarus; Institute of Genetics and Cytology of the National Academy of Sciences of Belarus, Minsk, Belarus; Institute of Genetics and Cytology of the National Academy of Sciences of Belarus, Minsk, Belarus; Institute of Genetics and Cytology of the National Academy of Sciences of Belarus, Minsk, Belarus

**Keywords:** barley, alloplasmic lines, chloroplast DNA, mitochondrial DNA, next generation sequencing (NGS), ячмень, аллоплазматические линии, хлоропластная ДНК, митохондриальная ДНК, высокопроизводительное секвенирование

## Abstract

Alloplasmic lines are a suitable model for studying molecular coevolution and interrelations between genetic systems of plant cells. Whole chloroplast (cp) and mitochondrial (mt) genome sequences were obtained by the MiSeq System (Illumina). Organelle DNA samples were prepared from a set of 12 alloplasmic barley lines with different cytoplasms of Hordeum vulgare ssp. spontaneum and H. vulgare ssp. vulgare, as well as from their paternal varieties. A bioinformatic approach for analysis of NGS data obtained on an organellar DNA mix has been developed and verified. A comparative study of Hordeum organelle genomes’ variability and disposition of polymorphic loci was conducted. Eight types of chloroplast DNA and 5 types of mitochondrial DNA were distinguished for the barley sample set examined. These results were compared with the previous data of a restriction fragment length polymorphism (RFLP) study of organelle DNAs for the same material. Formerly established data about a field evaluation of alloplasmic barley lines were revised in the light of information about organelle genomes gained after NGS. Totally 17 polymorphic loci were found at exons of chloroplast genomes. Seven of the SNPs were located in the genes of the Ndh complex. The nonsynonymous changes of nucleotides were detected in the matK, rpoC1, ndhK, ndhG and infA genes. Some of the SNPs detected are very similar in codon position and in the type of amino acid substitution to the places where RNA editing can occur. Thus, these results outline new perspectives for the future study of nuclear-cytoplasmic interactions in alloplasmic lines.

## Abstract (ru)

Аллоплазматические линии являются подходящей моделью для изучения молекулярной коэволюции и взаимодействий между генетическими системами растительной клетки. C использованием MiSeq System (Illumina) были определены полногеномные последовательности ДНК органелл клетки – хлоропластов и митохондрий. ДНК органелл выделена из 12 образцов коллекции аллоплазматических линий ячменя с цитоплазмами Hordeum vulgare ssp. spontaneum (H. spontaneum) и H. vulgare ssp. vulgare (H. vulgare), а также из сортов ячменя доноров ядра. Разработан и верифицирован подход к анализу результатов NGS смесей хлоропластной и митохондриальной ДНК для сборки новых полных сиквенсов пластидных и митохондриальных геномов H. vulgare и H. spontaneum. Проведено сравнительное изучение изменчивости геномов органелл, локализованы полиморфные участки. Выделено восемь типов хпДНК и пять типов мтДНК. Полученная информация сопоставлена с результатами предыдущих исследований этих же линий методом полиморфизма длин рестрикционных фрагментов ДНК органелл. На основании сравнения полногеномных последовательностей хпДНК и мтДНК аллоплазматических линий и сортов доноров ядерных геномов пересмотрены полученные для них ранее данные по формированию признаков, связанных с продуктивностью. Семнадцать полиморфных локусов обнаружено в экзонах пластидных генов. Семь из них расположены в генах Ndh комплекса. Несинонимические замены нуклеотидов идентифицированы в генах matK, rpoC1, ndhK, ndhG, infA. Вероятно, некоторые SNP являются точками, где происходит эдитинг, о чем свидетельствуют позиции замены в кодоне и тип аминокислотной замены. Проведенное исследо- вание открывает новые перспективы для изучения ядерно-цитоплазматических взаимодействий на при- мере аллоплазматических линий.

## Introduction

Barley is one of the most important cereals in the world, after wheat and rice. The history of its cultivation stretches from ancient times (Pankin, von Korf, 2017). Peculiar distribution of different nuclear and cytoplasmic gene loci of barley across localities occurred together with the process of migration of humans (Saisho, Purugganan, 2007). In parallel with the process of barley spreading from the centers of origin to different climatic zones, definite changes in nuclear and cytoplasmic genes have happened. This led to change in the interactions of the nucleus and cytoplasm, the establishment of new nuclearcytoplasmic relations.

Chloroplasts and mitochondria are essential organelles in plant cells and play an important role in sustaining life. The genomes of organelles have a number of properties that make them indispensable for studying in various areas of modern biology: a large number of copies per cell; relatively conservative sequences; lack of recombination; maternal inheritance; coding the proteins for photosynthesis and respiration; endosymbiotic origin (Danilenko, Davydenko, 2003; Daniell et al., 2016).

The efficiency of photosynthesis (the cooperative work of chloroplast and nuclear genes) and the functioning of the mitochondrial respiratory chain complexes (the joint work of different subunits of mitochondrial complexes coded by nDNA and mtDNA) basically determine the productivity of plants.

Among the suitable models for study of nuclear-cytoplasmic interactions are allo- and isoplasmic plant lines. Alloplasmic lines are usually created via hybridization when the nucleus from one plant (species or subspecies) are placed into the cytoplasm of another plant through substitution backcrossing. Evolutionarily established relations between nucleus and cytoplasm are disrupted at this case, and various consequencies could arise as the result. There is the big set of data about different effects of nuclear-cytoplasmic interactions. Wheat alloplasmic lines with the nucleus from Triticum aestivum and cytoplasm from different Aegilops and Triticum species were studied (Kihara, 1951; Fukasawa, 1959; Mukai et al., 1978; Maan, 1979; Tsunewaki, 1980, 1993). Barley alloplasmic lines were also created and thoroughly studied (Baturа et al., 1989; Krepak et al., 1996). Different manifestations of nuclear-cytoplasmic interactions were investigated with the help of the alloplasmic lines collections: the influence of plasmon on morphogenesis, photosynthesis and respiration, fertility, different stress conditions, transmission and recombination of nuclear genes of plants (Palilova, Sylkova, 1987; Nakamura et al., 1991; Sychjova et al., 1998; Goloenko et al., 2002; Tsunewaki et al., 2002, 2019).

NGS of the whole chloroplast (cp) and mitochondrial (mt) genomes from different matrices (either total cell DNA, or a mixture of organelle DNA, or pure plastid and mitochondrial DNA) allows to explore large number of samples simultaneously, to obtain qualitatively new comparative data about the variability of cytoplasmic genomes (Nock et al., 2011; Twyford, Ness, 2017). Especially the sequencing of pure organelle DNA templates – chloroplast or mitochondrial (or mixture of organelle DNAs), gained after organelle lysis appears to be the most promising for organelle genome studies. In this case numerous genomic and organelle DNA-like sequences from nucleus are drawn away from analysis facilitating the process of organelle genomes assembly.

Complete chloroplast and mitochondrial genome sequences are essential for realizing and reshaping the phylogenetic relationships between closely related taxa and for improving our understanding of the evolution of plant species (Gornicki et al., 2014; Givnish et al., 2018).

The comparative study of the plastid genomes variability in alloplasmic barley lines (differing in origin of the cytoplasm donor) and their euplasmic analogues were performed.

## Materials and methods

**Study material.** Three barley varieties Vezha, Roland, Visit as well as twelve alloplasmic lines created and maintained in the Lab of Cytoplasmic Inheritance (Institute of Genetics and Cytology, NAS Belarus) were used for organelle DNA isolation (Table 1).

**Table 1.**
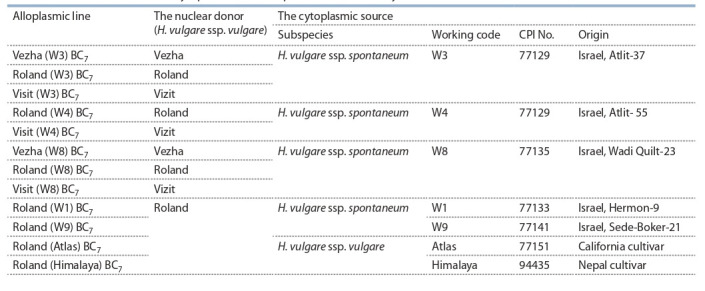
Sources of nucleus and cytoplasm at the alloplasmic lines of barley

We explored alloplasmic barley lines with cytoplasm of H. spontaneum and H. vulgare, as unfortunately we lack the original seeds of H. spontaneum and H. vulgare donors of cytoplasm (maternal parent). This plant material was created in 1990th and then maintained by self pollinating.

The maternal inheritance of organelle genomes is well documented in angiosperms, and considered as one of the laws of cytoplasmic inheritance (Birky, 2008), though very seldom occasional paternal transmission can occur (Reboud, Zeyl, 1994). We considered the plasmon of definite alloplasmic line is equivalent to the original plasmon of corresponding wild H. spontaneum, H. vulgare. Moreover, one of the recent studies of alloplasmic lines in Triticum-Aegilops complex demonstrated the stability of organellar DNA characteristics between native euplasmic plants and alloplasmic line, indicating generally the usefulness of organellar genome in tracing the maternal lineage of species (Tsunewaki et al., 2019).

The whole sequences of H. vulgare chloroplast (NC_008590, Saski et al., 2007) and mitochondrial (AP017301, Hisano et al., 2016) genomes accessible in NCBI nucleotide database (GenBank) were used as references for assembly of new barley organelle genomes.

**DNA extraction.** Organelles were extracted from 7 days seedlings. The fraction of organelles was obtained by differential centrifugation (Triboush et al., 1998). Chloroplast and mitochondrial DNA were obtained by lysis of isolated organelles with subsequent phenol-chloroform deproteinization. This approach allowed simultaneous sequencing of both genomes from each sample. The quality and concentration of DNA was evaluated after 0.8 % agarose gel electrophoresis and at NanoDrop 8000 spectophotometer (Thermo Fisher Scientific).

**NGS.** The NGS was perfomed on the MiSeq System (Illumina Inc., SanDiego, CA, USA), library preparation kit NexteraXT, MiSeq Reagent Kit v3, read lengths 300 bp.

**NGS data processing**. The raw data were exported for the primary analysis. The algorithm of sequencing data processing included the following steps: trimming of raw reads (Trimmomatic; Bolger et al., 2014); aligning reads to the “double” reference, containing full sequences of chloroplast and mitochondrial barley genomes (Bowtie2, http://bowtiebio.sourceforge.net/bowtie2/index.shtml); obtaining mapping statistics (bash scripts, BCFtools, https://samtools.github.io/bcftools/bcftools.html); alignment visualization (Tablet; Milne et al., 2013); generating VCF files (Samtools; Li et al., 2009); filtering VCF files (VCFlib, https://bio.tools/vcflib). The algorithm was tested on artificial Illumina reads synthesized using the ART program. Ultimately, VCF files containing all polymorphic loci of the chloroplast and mitochondrial genomes were obtained (Makarevich et al., 2018).

**Comparative analysis of cp genomes.** The VCF files for cp genomes of all studied samples as well as accessible complete cp genome sequences of H. vulgare ssp. vulgare (NC_008590) and H. vulgare ssp. spontaneum (KC912688, KC912689) taken from the NCBI GenBank database were involved in whole-genome comparison analysis. To visually display the phylogenetic relationships between the studied samples and divide them into groups by the types of chloroplast genomes, the maximum parsimony-cladogram was constructed on the base of all founded SNPs and INDELs. Complete chloroplast genome sequences of H. jubatum (KM974741) and H. bulbosum (KY636105) available in NCBI GenBank acted as outgroups. The cladogram was built in Excel (Version 14.0.6112.5000) with searching for homologous regions in the outgroup genomes using the SnapGene V.4.3.10.

## Results and discussion

15 chloroplast and 15 mitochondrial genome sequences were obtained after NGS (Illumina, MiSeq) of organelle DNA samples (plastid + mitochondrial fraction). Bioinformatics’ approaches have been optimized for the processing of the “raw” data after sequencing the mixture of cp and mtDNA. The comparative analysis of the obtained sequences as well as available in NCBI nucleotide database (GenBank) was carried out, that promoted the assessment of the total level of sequence variation between samples of the same species, defined the regions, where changes more often occur.

Generally, 103 polymorphic loci of cpDNA were revealed after comparison analysis of 15 obtained new full organelle sequences and 4 sequences from GenBank (see Materials and methods). Among them 78 differences for the alloplasmic lines and barley paternal varieties were detected: 56 SNP (39 of them at introns of various genes, 17 – at exons (see Table 4)), 14 SSR loci and 8 indels.

**Table 4.**
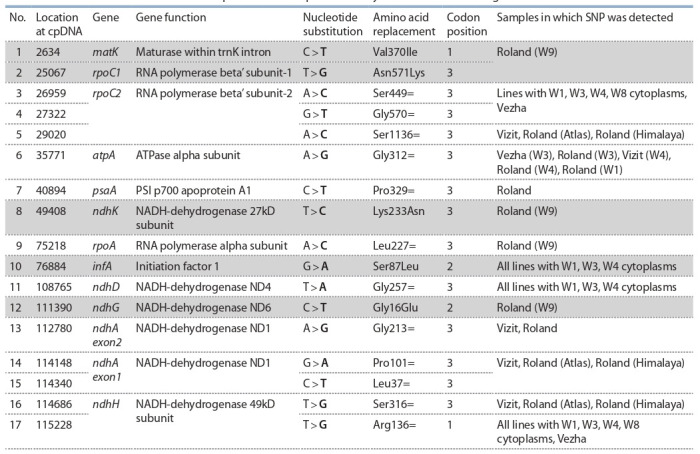
List of SNP detected at exons of cpDNA after comparative analysis of obtained whole genomes Note: The grey filling in the table – marked SNP, where nonsynonymous changes of nucleotides occurred.

The cladogram for the complete chloroplast genomes of barley was constructed on the base of polymorphic loci found, except for the SSR regions (see the Figure). It displays the diversity of chloroplast genomes of H. vulgare and H. spontaneum, which allows to subdivide the plasmotypes in the study set of barley (see below Table 2).

**Fig. 1.**
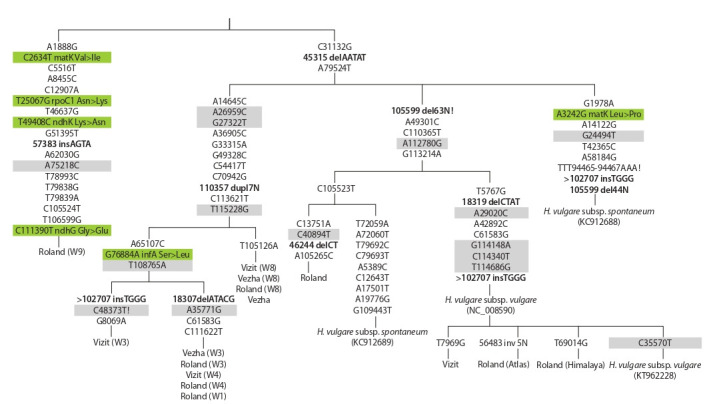
The cladogram for the complete chloroplast genomes of barley (based on variability regions). Maximum parsimony-cladogram was constructed on the base of founded SNPs and INDELs. Chloroplast genome sequences of H. jubatum (KM974741) and H. bulbosum (KY636105) available in NCBI GenBank were used as outgroups. The polymorphic loci are given according to reference H. vulgare (NC_008590). Highlighted in green – synonymous nucleotide substitutions, grey – nonsynonymous substitutions. Indels are written in bold. Exclamation mark (!) – reverse replacements , “>” – parallel mutations.

**Table 2.**
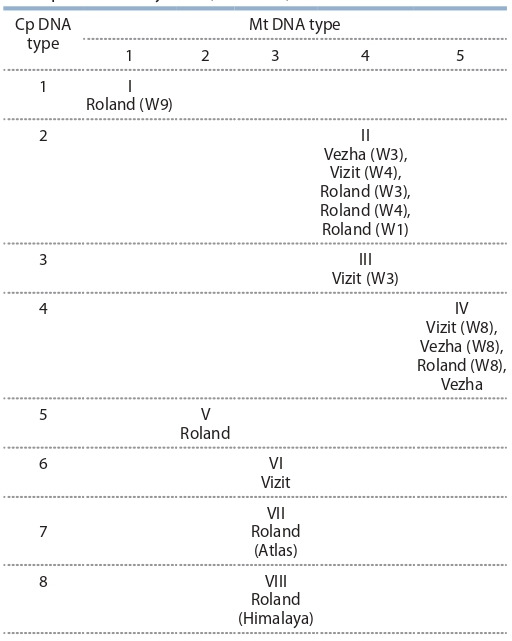
The specific combinations of chloroplast and mitochondrial DNA types in the collection of alloplasmic barley lines (NGS data)

20 loci of mtDNA variability were identified. The level of sequence variation was much lower compared to chloroplast DNA. Only two types of changes were detected – point nucleotide substitutions (19) and insertion (1). All the indels and the most significant SNP were checked by Sanger sequencing to verify the assembly of both genomes.

Qualitatively new data about the variability of Hordeum organelle genomes and disposition of polymorphic loci were obtained after NGS. It turned out to be higher than described in earlier reports based on RFLP data or SSR analysis of relatively few loci (Neale et al., 1988; Provan et al., 1999; Russell et al., 2003; Lukhanina et al., 2006; Sipahi et al., 2013).

**Differentiation of the alloplasmic lines collection according various molecular methods**

As a result of NGS study, eight types of chloroplast DNA and five types of mitochondrial DNA were distinguished for the examined barley sample set (Table 2). We were able to highlight eight plasmotypes totally.

Earlier in 1984 the same set of cytoplasms as well as some others: W1, W3, W4, W5, W7, W8, W9, W10, Atlas, Himalaya, L1, L2 – were studied via restriction of cpDNA with 4 endonucleases BamHI, BclI, EcoRI, HindIII (Clegg et al., 1984). Five different chloroplast RFLP patterns were detected. Afterwards mtDNA endonuclease digestion was performed for 12 alloplasmic lines with the above mentioned cytoplasms and nucleus from barley variety Vezha. Four groups of mtDNA types were detected (Sychjova et al., 1998). Combining these results, 9 specific plasmotypes were distinguished (Table 3).

**Table 3.**
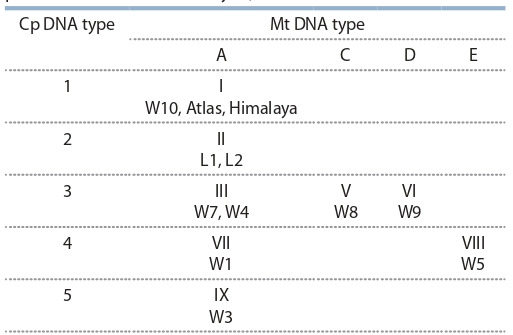
The specific combinations of chloroplast and mitochondrial DNA types in the collection of alloplasmic barley lines (according to previously published data of RFLP analysis)

NGS data for chloroplast genome (this work) and earlier obtained RFLP data (Clegg et al., 1984) were compared. RFLP analysis having quite low resolution was not able to show all the differences between samples. So, we expected that sequencing of full chloroplast and mitochondrial genomes of alloplasmic lines will shed light on the organelle genome variability of this collection.

Specific motifs for BamHI, BclI, EcoRI, HindIII endonucleases were determined in obtained full chloroplast genomes (SnapGene V.4.3.10). Despite many variability positions between samples detected by NGS (see the Figure), they mainly didn’t affect the enzyme cutting sites (c. s.). All samples bear the same endonuclease digestion points for BamHI (54 c. s.), BclI (53 c. s.), EcoRI (97 c. s.), HindIII (40 c. s.). Just only G33315A substitution led to appearance the new cutting site for BclI in W1, W3, W4, W8 and Vezha cytoplasm (BclI is Dam methylation-sensitive enzyme, so it could influence the RFLP results). All together they bear 54 BclI sites.

The Roland (W9) appeared to be especially unique, because only this line had four new SNP: A1888G (created the new digestion site for HindII ), T25067C, T79838G and T79839A (that promote the emergence of two new EcoRI cutting points). Thus Roland (W9) bears 41 HindIII and 99 EcoRI cleavage points.

We tried to collate the types of cytoplasms obtained by two different methodological approaches – RFLP and NGS sequencing (see the Figure and Table 2, 3).

Roland (Atlas) and Roland (Himalaya) refer to the same plasmotype according to RFLP data, but NGS differentiated it.

According to Clegg (Clegg et al., 1984), W3 and W4 belong to different RFLP because of different cpDNA types and one common mitochondrial. However, the NGS study combined them in one group, with one exeption – Vizit (W3). Note an interesting fact – W3 and W4 lines originated from one locality Atlit at Israel and they are most likely closer genetically than other lines studied. Sequencing data put alloplasmic lines with W3 and W4 cytoplasm at one subcluster. So, Vezha (W3), Roland (W3), Roland (W4), Vezha (W4) are identical for all polymorphic loci and Vizit (W3) is very similar to other alloplasmic lines with W3 cytoplasm, bearing variability only at 7 polymorphic loci.

Alloplasmic lines with W8 cytoplasm formed separate group according to both methods.

Roland (W9) line was placed into the special RFLP group; according to NGS data, this line has more than 20 specific SNP, thus it appears to be remote from all other lines studied (see the Figure).

**SNP detected at chloroplast genome sequences, their possible importance for plants**

Only single nucleotide substitutions were revealed at exons of chloroplast genes, 12 of them were synonymous: rpoA, rpoC1, rpoC2, ndhA, ndhD, ndhG, ndhH, ndhK, atpA, psaA genes. The 5 nonsynonymous changes of nucleotides were detected in matK, rpoC1, ndhK, ndhG and infA genes (Table 4).

Some of SNP detected are very similar (position in codon and also the type of amino acid substitution) to the places where RNA editing can occur. Editing usually appeared to be C-U replacement at the mRNA level, it corrects the “wrong” nucleotide at the DNA level. It leads to restoration of phylogenetically conservative amino acid sequences of the proteins (Maier et al., 1996; Danilenko, Davydenko, 2003). Editing is the important posttranscriptional control of the gene expression. It is necessary for plastids functioning and plants survival (Takenaka et al., 2013). Nucleotide substitutions in matK 2634 С > T (p.Val370Ile), ndhK 40894 C > T (p.Lys233Asn), infA 76884 G > A (p.Ser87Leu), ndhG 111390 C > T (p.Gly16Glu), ndhA exon2 112780 A > G (p.Gly213=), ndhA exon1 114148 G > A (p.Pro101=), ndhA exon1 114340 C > T (p.Leu37=) possibly mark positions where editing could take place.

According to Table 4, SNP at cpDNA 76884 nucleotide position resulted in amino acid substitution p.Ser87Leu at infA gene for lines Hordeum vulgare subsp. spontaneum with W3 and W4 cytoplasm. infA is the factor 1 of translation initiation (IF1) encoded in chloroplast. It is highly conserved and universal in all living organisms (Roll-Mecak et al., 2001). Remarkably we found polymorphic loci at infA gene, position 76884 G > A of cpDNA. This SNP occurred at the second position of codon, usual position for editing (87 % of all edited sites – the second nucleotide positions of triplets encoding amino acids). The barley with cytoplasms W3, W4, and W1 bear A (or T at opposite DNA strand) and they needn’t correction C > T at the RNA level (editing), but all the other samples studied possess C and it can be corrected by editing machinery. This substitution will lead to Ser>Leu change – most common conversion during editing (Tsudzuki et al., 2001). Some further work to support this idea and to determine functional peculiarities of this event is needed.

About half of SNP (7 out of 17) are located at the genes of NADH complex. Chloroplast DNA of barley encodes 11 Ndh genes. Of note that these genes are quite variable in different taxons and still remain between the relatively few number of genes retained at cpDNA of higher plants in comparison with genome of prokaryotic Cyanobacteria ancestor. They have been lost in Gnetales, pines, Erodium species, some parasitic plants, endowing their characteristic cpDNA features (Peredo et al., 2013; Sabater, 2018). Nevertheless, most land photosynthetic plants contain these genes. It is considered, that they are important for adaptation of plants to photosynthesis (Martin, Sabater, 2010; Shikanai, 2016). Ndh complex function is necessary to optimize photophosphorylation rates under different stress conditions (Rumeau et al., 2007; Martin, Sabater, 2010).

Nearly 50 % of editing sites of flowering plants concentrate at the ndh group of genes (Martin, Sabater, 2010). We found loci of variability at five of them. Possibly nucleotide substitution C > T at 111390 position of cpDNA resulted in amino acid change Gly16Glu, can be the place of editing event which needs future verification.

The productivity characters of substituted barley lines with five different nuclear genomes and six plasmotypes were thoroughly tested in field conditions for several years (Goloenko et al., 2000). The direct correlation between productivity and differences in the structure of organelle genomes was not found. The effects of cytoplasm substitution on the economically valuable traits of plants varied greatly depending on nuclear genome, significant impact for various traits was revealed for definite nuclear-cytoplasmic combinations. Nevertheless, two groups of cytoplasms with minimal – W4, W8, and highest influence on productivity – A, L1, W3, W5 were defined on the basis of comparative analysis.

## Conclusion

We have got detailed information about sequences of barley organelle genomes of set of alloplasmic lines. At present we cannot precisely foresee which cytoplasm type gives definite phenotypic effect. The results prove that interaction of definite nucleus with specific organelle genome in every case can be unique. Nevertheless, each line was marked and its peculiar features were defined. It represents the new type of starting material for prospective studies in breeding and molecular genetics in Poaceae.

Furthermore, alloplasmic lines appeared to be the promising material for the study of molecular coevolution between genetic systems of plant cell. If editing really occurs at some loci of organelle genes of definite barley cytoplasms it needs special transfactors, at least PLS-type PPR proteins (encoded in nucleus). These proteins are highly specific for their RNA targets. If we combine in alloplasmic lines cpDNA which needs editing and nucleus where transfactor for this event is not encoded, how could plants overcome this inconsistency? Another case – the cytoplasm loci need no editing, but transfactor is encoded in nucleus. Amazingly, phylogenetic comparison of editing sites and corresponding PPR proteins leads to the very interesting fact: if conversion C to T at the DNA level takes place, no need in editing exists and PPR protein can gradually disappear from the nucleus of this taxon. Moreover, as revealed for DOT4 PPR protein – the loss of editing target site through C to T conversion allowed DOT4 in Poaceae to adapt for new function. Also some cases were described where the target sequences for editing exist, but no corresponding PPR is encoded by nucleus (Hein et al., 2019).

Thus, the field for further studies of nuclear-cytoplasmic cooperation is quite large. Possibly editing (PLS-PPRs) or other processes and their players both from nucleus and cytoplasm are involved, that bring us to discovery of fine mechanisms of different cell genetic systems interaction.

## Conflict of interest

The authors declare no conflict of interest.
